# Rare clinical concomitancy in female patient: still syndrome and omeprazole pharmacodermy - case report

**DOI:** 10.1186/1939-4551-8-S1-A238

**Published:** 2015-04-08

**Authors:** Silvia Maria Tintori, Willian Luiz Rombaldo, Daniel Lopes Aires, Mário Henrique Verussa

**Affiliations:** 1 State University of Maringá, Maringá, Paraná 87083240, Brazil

## Background

Adverse drug reactions account for 11.5% of hospitals admissions (MASTROIANNI et al). Among the drugs involved in the etiology of these reactions, Omeprazole is one of the most frequent. Although this approach unusual conditions, we can highlight the Still syndrome, systemic inflammatory disease (OWLIA MB, MEHRPOOR G). By coursing with similar dermatological symptoms, the physician must have an active critical thinking and pharmacological knowledge determined to establish the differential diagnosis of the disease as the presenting symptoms.

## Methods

In this case report, we describe drug eruption associated with Omeprazole in a patient previously diagnosed with Still Syndrome.

## Results

After extensive medical history taken by the allergist, the patient revealed chronic use of Omeprazole for over ten years, so far not reported for the same. Thus, the suspension was indicated in the use of drug rash with complete remission. Endocrine, skin, nerve and muscle disorders, although rare, may also occur in the patient who makes use of such drug. In this sense, the cutaneous reactions of the type erythematosus rash are symptoms that, although rare, may also manifest in patients, as reported in this case. Diseases like Still syndrome can mimic dermatological picture with the same cutaneous manifestations (OWLIA MB, MEHRPOOR G), which contributed to the difficult elucidation of this urticaria and angioedema diagnosis.

## Conclusions

The resolution of the urticária and angioedema presented by the patient was only possible thanks to an accurate anamnesis with her, added to the fact of medical knowledge about the medicinal drug (reaction to Omeprazol), including those more rare, which ratifies the need for critical thinking reactions and pharmacological knowledge of the professional in your work routine.

